# Association between carotid atherosclerosis and different subtypes of hypertension in adult populations: A multiethnic study in Xinjiang, China

**DOI:** 10.1371/journal.pone.0171791

**Published:** 2017-02-15

**Authors:** Yun Wu, Fen Liu, Dilare Adi, Yi-Ning Yang, Xiang Xie, Xiao-Mei Li, Xiang Ma, Zhen-Yan Fu, Ying Huang, Bang-Dang Chen, Chun-Fang Shan, Yi-Tong Ma

**Affiliations:** 1 Department of Cardiology, First Affiliated Hospital of Xinjiang Medical University, Urumqi, P.R. China; 2 Department of General Practice, First Affiliated Hospital of Xinjiang Medical University, Urumqi, P.R. China; 3 Xinjiang Key Laboratory of Cardiovascular Disease Research, First Affiliated Hospital of Xinjiang Medical University, Urumqi, P.R. China; Nagoya University, JAPAN

## Abstract

**Background:**

Ethnic differences in non-invasive measurements of carotid atherosclerosis are being increasingly reported, but the association between carotid atherosclerosis and different subtypes of hypertension in adult populations is not fully understood in different ethnicities. We aimed to investigate the association of carotid atherosclerosis with different subtypes of hypertension in different ethnicities in Xinjiang, a northwestern province in China.

**Methods:**

A total of 14,618 participants (5,757 Hans, 4,767 Uygurs, and 4,094 Kazakhs) from 26 villages of seven cities in Xinjiang were randomly selected from the Cardiovascular Risk Survey conducted during 2007 and 2010. A standard questionnaire, a physical examination and biochemical tests were employed.

**Results:**

The mean common carotid intima-media thickness (CIMT) for the 14,618 participants was 0.86±0.003 mm. The CIMT gradually increased with age. Men (0.92±0.005 mm) had a higher CIMT than women (0.81±0.004 mm). The Uygur participants (0.82±0.006 mm) had a lower CIMT than the Han (0.88±0.005 mm) and Kazakh participants (0.88±0.005 mm). The overall prevalences of carotid intimal thickening and carotid plaques were 12.4% and 9.7%, respectively. The prevalence of CIMT varied for the different subtypes of hypertension. Multivariate logistic regression analysis showed different risk factors for abnormal CIMT in different ethnicities. The associations between abnormal CIMT and the different subtypes of hypertension within different ethnic backgrounds were also different. The risk factors for abnormal CIMT included systolic-diastolic hypertension (SDH) in Han participants (OR: 1.323, 95% CI: 1.100–1.590), SDH (OR: 1.426, 95% CI: 1.160–1.753) and isolated-systolic hypertension (ISH) (OR: 1.844, 95% CI: 1.470–2.313) in Uygur participants, and isolated-diastolic hypertension (IDH) (OR: 1.536, 95% CI: 1.170–2.016) in Kazakh participants.

**Conclusion:**

There was an ethnic difference in the prevalence of abnormal CIMT in Xinjiang, a northwestern province in China. The associations between abnormal CIMT and the subtypes of hypertension varied among the different ethnic groups. Among the studied populations, Han participants with SDH, Uygur participants with SDH and ISH, and Kazakh with IDH were more likely to suffer carotid atherosclerosis than those with other subtypes of hypertension. Participants with different ethnic backgrounds had different sets of risk factors for abnormal CIMT.

## Introduction

Atherosclerosis, a diffuse vascular disease, is a process in which fatty deposits, inflammatory cells, and scar tissue build up within the walls of arteries; this process is characteristically silent until it progresses to critical stenosis, thrombosis, aneurysm, or embolus supervenes in the lumens [1]. Thus, atherosclerosis is an underlying cause of most clinical cardiovascular and cerebrovascular events. Early identification of the risk factors for atherosclerosis could lead to more positive lifestyle modifications and medical treatment to prevent clinical manifestations of atherosclerosis such as myocardial infarction, stroke, or renal failure [2]. Carotid arteries are important conduits that connect the heart and the brain and are thought to develop atherosclerosis earlier than coronary arteries. Carotid intima-media thickening and carotid plaques, which are measurement parameters of carotid atherosclerosis, can be measured by B-mode ultrasonography. Furthermore, carotid intima thickening is thought to be an early predictor of atherosclerosis because it precedes the development of true atherosclerotic plaques. In addition, some studies have shown that carotid intima-media thickness (CIMT) measurements by B-mode ultrasonography may provide an independent assessment of coronary risk [3–5].

Among many risk factors, hypertension plays an important role in the development of atherosclerosis and is a major risk factor for cardiovascular disease and stroke. Fluctuations in blood pressure affect vascular endothelial cell morphology, structure and function and vascular wall permeability, which can contribute to the accumulation of fat and cells [6]. In a recent study, the prevalence of hypertension was up to 44.3% in China and 37% in the western region of China. [7]. Previous studies have shown that hypertension is significantly associated with the progression of carotid atherosclerosis [8–10]. However, the association between carotid atherosclerosis and hypertension in multiethnic adult populations is not fully understood in Xinjiang, China.

In this study, we focused on the relationship between carotid atherosclerosis and hypertension in the Han, Uygur and Kazakh populations in Xinjiang. This study will help us to investigate the following: 1) the prevalence of carotid atherosclerosis in the different ethnic populations in Xinjiang, a northwestern province in China; 2) the associations between CIMT and different subtypes of hypertension in different ethnic backgrounds; 3) the degree to which these differences might be explained by ethnicity-related CIMT risk factors and other covariates.

## Methods

### Ethics statement

This study was approved by the Ethics Committee of the First Affiliated Hospital of Xinjiang Medical University (Xinjiang, China). It was conducted in accordance with the standards of the Declaration of Helsinki. Signed informed consent was obtained from each participant before entering this study.

### Subjects

The Cardiovascular Risk Survey (CRS) was designed to investigate the prevalence and incidence of cardiovascular diseases and their risk factors and to determine the genetic and environmental contributions to atherosclerosis and coronary artery disease in the Chinese Han, Uygur, and Kazakh populations in Xinjiang, China. Adult participants, aged ≥ 35 years old, were initially recruited and examined from 2007–2010 in seven cities in Xinjiang, in the northwestern part of China. A detailed description of the study population and methods has been previously reported [11, 12]. In brief, 14,618 participants (5,757 Hans, 4,767 Uygurs, and 4,094 Kazakhs) were randomly selected from 26 villages of seven cities in Xinjiang. Patients with a previous cardiovascular event such as myocardial infarction or stroke as well as heart failure were excluded. Moreover, patients subjected to previous anti-atherosclerosis therapy, such as statins and aspirin, and patients with incomplete data (677 Hans, 605 Uygurs, 490 Kazakhs) were excluded from the analysis.

### Data collection and biochemical analysis

This survey included a questionnaire, a physical examination and biochemical tests. Cardiovascular physicians underwent standardized training before they performed the medical examination and inquiry. The questionnaire was used to collect demographic characteristics and medical histories, as previously described [11–13]. Height and body weight were measured using standard methods. Smoking and drinking conditions were self-reported. To assess the smoking or drinking status of the participants, we asked the following question: “Prior to this study, have you ever smoked or drunk alcoholic beverages?” Those who answered “no” to this question were classified as non-smoking or non-drinking.

After an overnight fast of 12 h, venous blood samples were collected from all participants and processed to obtain plasma within 4 h in the examination centers of local hospitals in the participants’ residential area. Serum concentrations of triglyceride (TG), total cholesterol (TC), high-density lipoprotein cholesterol (HDL-c), low-density lipoprotein cholesterol (LDL-c), fasting blood glucose (FBG), uric acid (UA), blood urea nitrogen (BUN) and creatinine (Cr) were measured with chemical analysis equipment (Dimension AR/AVL Clinical Chemistry System, Newark, NJ) at the Clinical Laboratory Department of the First Affiliated Hospital of Xinjiang Medical University, as previously described [11–13].

### Blood pressure measurement

Blood pressure (BP) levels were measured using a standard protocol, as previously described [13]. After a 15-min resting period, sitting blood pressure was measured by trained staff members using mercury sphygmomanometers. Subjects were required to refrain from smoking or consuming caffeine. Two measurements were performed consecutively at 15-min intervals, and the mean values of systolic blood pressure (SBP) and diastolic blood pressure (DBP) were calculated for analysis. If the first two measurements in either the SBP or DBP differed by more than 5 mmHg, an additional measurement was taken.

### Definition of hypertension

Hypertension was defined in accordance with the Joint National Committee guidelines (JNC 8) as follows [14]: SBP≥140 mmHg, DBP≥90 mmHg, taking antihypertensive medicine, or having been told at least twice by a physician or other health-care professional that one has HBP. Participants without anti-hypertensive treatment were further grouped as follows: isolated diastolic hypertension (IDH) with SBP<140 mmHg and DBP≥90 mmHg; isolated systolic hypertension (ISH) with SBP≥140 mmHg and DBP <90 mmHg; and systolic-diastolic hypertension (SDH) with SBP≥140 mmHg and DBP≥90 mmHg [15]. Participants that were undergoing anti-hypertensive treatment were categorized according to the subtypes initially presented and recorded in their medical history.

### CIMT measurements

The carotid arteries were evaluated by a single specialist who was blinded to the subject details with a 7.5-MHz linear-type B-mode probe (Siemens, Berlin, Germany). The bilateral carotid arteries of the participants were scanned in the supine position with the neck hyperextended. Images of the common carotid artery (CCA) were used to evaluate the CIMT. The CIMT was determined as the distance from the media-adventitia interface to the intima-lumen interface on the far wall in a region free of plaques. Between the carotid bulb origin and a point 10 mm proximal to the common carotid artery on the longitudinal view (10 mm in length), we performed three measurements to determine the maximal CIMT of the CCA. Carotid intimal thickening was defined as 1.0≤CIMT<1.5 mm, and carotid plaques were defined as a discrete focal-wall thickening ≥1.5 mm or focal thickening at least 50% greater than the surrounding CIMT [12,16].

### Data management and statistical analysis

All data from the questionnaire were double-entered and cross-validated using EpiData version 3.1 (EpiData Association, Odense, Denmark). Statistical analyses were performed using SPSS version 17.0 (SPSS Institute, Chicago, IL, USA). Continuous variables are expressed as the mean±SD or mean±SEM, categorical variables are expressed as percentages, and exploratory data analysis was performed using descriptive measures. A chi-square test (χ2) was used to detect associations in the categorical data and to identify differences in prevalence. Potential risk factors for atherosclerosis were analyzed using a multivariate unconditional logistic regression. A value of P<0.05 was regarded as statistically significant.

## Results

### General characteristics of the study population

In this study, 14,618 individuals were enrolled, including Han (5,757), Uygur (4,767) and Kazakh (4,094) ethnic groups. General characteristics of the study participants have been previously described [13]. In brief, besides the level of LDL-c, there were significant differences in age, BMI, smoking, drinking, SBP, DBP, and other biochemical parameters. The Kazakh participants, who had a mean age of 48.63±11.69 years (P<0.05), were younger than the Han and Uygur participants. The prevalence of hypertension was 40.1% in all participants and varied greatly among the ethnic groups (38.9%, 32.9% and 50.3% in Han, Uygur and Kazakh, respectively). The Kazakh participants had the highest BP values, which were significantly higher than those in the Han and Uygur participants (both P<0.05). The treatment rate of hypertension was 16.6% in the total population. (Table A in S1 File)

### CIMT based on gender, ethnicity and age in the total study group

**Fig 1:**
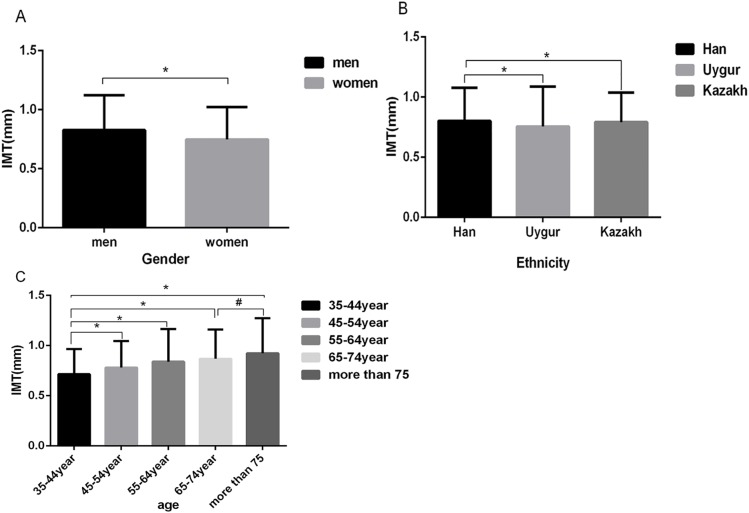
CIMT by gender, ethnicity and age in the total participant group. **A.** Overall gender difference in CIMT. *P<0.001 vs. men. **B.** Overall ethnic difference in CIMT. *P<0.001 vs. Han. **C.** Overall difference of CIMT in different age groups. *P<0.001 vs. the 35- to 44-year-old group. ^#^ P>0.05 vs. the 65- to 74-year-old group.

Among the 14,618 participants, the mean common CIMT was 0.86±0.003 mm. When stratified by gender, the CIMT was higher in men (0.92±0.005 mm) than in women (0.81±0.004 mm) (Fig 1A). When stratified by ethnicity, the Uygur participants had the lowest CIMT (0.82±0.006 mm) among the three ethnic groups (P<0.001), and there was no significant difference between the Han (0.88±0.005 mm) and Kazakh participants (0.88±0.005 mm) (P>0.05) (Fig 1B). When stratified by age, the CIMT was lowest in the 35- to 44-year-old group (0.76±0.004 mm) and highest in the 65- to 74-year-old group (1.00±0.010 mm) and the >75-year-old group (1.00±0.020 mm). We employed a trend test to examine the association between CIMT and age (F = 19.44, P<0.001). CIMT gradually increased with age (Fig 1C); however, there was no significant difference between the 65- to 74-year-old group and the >75-year-old group (P>0.05).

Furthermore, the overall prevalence of carotid intimal thickening was 12.4% and the prevalence of carotid plaques was 9.7%. The prevalence of carotid intimal thickening and plaque stratified by gender, age and ethnicity was investigated (Table 1). When stratified by gender, the prevalence of carotid intimal thickening was 14.7% in men and 10.4% in women, and the prevalence of carotid plaques was 12.2% in men and 7.4% in women, indicating a significant difference between genders (P<0.001). When stratified by age, the highest prevalence of carotid intimal thickening was in the 65- to 74-year-old group (16.7%) and the lowest prevalence was in the 35- to 44-year-old group (9.2%); the highest prevalence of carotid plaques was in the >75-year-old group (18.3%) and the lowest prevalence was in the 35- to 44-year-old group (5.2%) (P<0.001). The prevalence of carotid intimal thickening in the different ethnic groups with hypertension also varied significantly (P<0.001). Furthermore, to exclude the influence of age on atherosclerosis in the different ethnic groups, we standardized the results by age, which yielded similar results regarding the prevalence of carotid intima thickening and carotid plaques (P<0.001).

**Table 1:** CIMT measurements (n, %) by sex, age and ethnicity.

	Normal CIMT (n, %)	Carotid Intimal Thickening (n, %)	Carotid Plaques (n, %)	Pearson χ^2^	p
**Sex**				180.234	<0.001
Male	4985 (73.1%)	1001 (14.7%)	833 (12.2%)		
Female	6412 (82.2%)	809 (10.4%)	578 (7.4%)		
**Age**				507.883	<0.001
35~44 years	4643 (85.6%)	500 (9.2%)	282 (5.2%)		
45~54 years	2965 (78.9%)	484 (12.9%)	310 (8.2%)		
55~64 years	2148 (73.3%)	413 (14.1%)	371 (12.7%)		
65~74 years	1302 (65.5%)	333 (16.7%)	354 (17.8%)		
> 75 years	339 (66.1%)	80 (15.6%)	94 (18.3%)		
**Ethnicity**				108.99	<0.001
Han	4429 (76.9%)	750 (13.0%)	578 (10.0%)		
Uygur	3776 (79.2%)	445 (9.3%)	546 (11.5%)		
Kazakh	3192 (78.0%)	615 (15.0%)	287 (7.0%)		
**After age standardization**					
Han	77.33%	13.5%	10.97%	80.80	<0.001
Uygur	87.78%	8.19%	8.99%		
Kazakh	78.47%	14.86%	6.66%		

CIMT, carotid intima-media thickness. Normal CIMT, CIMT<1.0 mm; Carotid Intimal Thickening, 1.0≤CIMT<1.5 mm; Carotid Plaques; CIMT≥1.5 mm or focal thickening at least 50% greater than the surrounding CIMT.

### Associations of CIMT with blood pressure

In this study, we observed an association between CIMT and blood pressure in the total population. We found a significant correlation between CIMT and blood pressure level based on Pearson’s correlation, including the levels of SBP (r = 0.170, P<0.001) and DBP (r = 0.137, P<0.001). Additionally, we observed an association of CIMT with blood pressure in the different ethnic groups. We found that the CIMT was significantly correlated with SBP (Han, r = 0.183; Uygur, r = 0.204; Kazakh, r = 0.105; all, P<0.001) and DBP (Han, r = 0.146; Uygur, r = 0.137; Kazakh, r = 0.100; all, P<0.001) for the three ethnicities. These results imply that the CIMT increases with increasing SBP or DBP in the different ethnic groups.

### Associations of CIMT with different subtypes of hypertension based on ethnicity

To identify whether different subtypes of hypertension have a different impact on CIMT, we investigated the associations between CIMT and the subtypes of hypertension in the 3 ethnic groups (Table 2). From these results, we found that different subtypes of hypertension were strongly associated with changes in CIMT and that there were significant variations among the different ethnic groups (P<0.001).

**Table 2:** CIMT measurements (n, %) by ethnic groups with different subtypes of hypertension.

	Normal CIMT (n, %)	Carotid Intimal Thickening (n, %)	Carotid Plaques (n, %)	Pearson χ^2^	p
**All**				227.232	<0.001
Normal BP	6991 (82.0%)	907 (10.6%)	627 (7.4%)		
IDH	891 (74.4%)	191 (16.0%)	115 (9.6%)		
SDH	2248 (71.4%)	478 (15.2%)	423 (13.4%)		
ISH	1267 (72.5%)	234 (13.4%)	246 (14.1%)		
**Han**				97.936	<0.001
Normal BP	2662 (80.6%)	402 (12.2%)	239 (7.2%)		
IDH	462 (74.2%)	94 (15.1%)	67 (10.8%)		
SDH	771 (69.1%)	159 (14.3%)	185 (16.6%)		
ISH	534 (74.6%)	95 (13.3%)	87 (12.2%)		
**Uygur**				163.507	<0.001
Normal BP	2654 (84.1%)	217 (6.9%)	284 (9.0%)		
IDH	167 (77.3%)	31 (14.4%)	18 (8.3%)		
SDH	594 (71.1%)	113 (13.5%)	129 (15.4%)		
ISH	361 (64.5%)	84 (15.0%)	115 (20.5%)		
**Kazakh**				41.689	<0.001
Normal BP	1675 (81.0%)	288 (13.9%)	104 (5.0%)		
IDH	262 (73.2%)	66 (18.4%)	30 (8.4%)		
SDH	883 (73.7%)	206 (17.2%)	109 (9.1%)		
ISH	372 (79.0%)	55 (11.7%)	44 (9.3%)		

CIMT, carotid intima-media thickness; BP, blood pressure; IDH, isolated diastolic hypertension; SDH, systolic-diastolic hypertension; ISH, isolated systolic hypertension.

### Identification of abnormal CIMT risk factors

To identify the potential risk factors for carotid atherosclerosis, we compared multiple factors, including carotid intimal thickening and plaque, between the abnormal-CIMT participants and normal CIMT participants using multivariate unconditional logistic regression analysis; the statistical results are presented in Table 3. The risk factors included gender, age, ethnicity, smoking status, drinking status, BMI, diabetes status, as well as subtypes of hypertension, anti-hypertension therapy, blood lipid parameters, BUN, Cr and UA.

**Table 3:** Multivariate unconditional logistic regression analysis of the risk factors for abnormal CIMT in study participants.

Factor	B	S.E.	Wald	df	P	OR	95% CI
Gender	-0.351	0.056	39.770	1	<0.001	0.704	0.631–0.785
Age			237.573	4	<0.001		
35–44						1	
45–54	0.393	0.060	43.058	1	<0.001	1.481	1.317–1.665
55–64	0.680	0.063	116.829	1	<0.001	1.975	1.746–2.234
65–74	1.003	0.070	204.424	1	<0.001	2.727	2.377–3.129
>75	0.919	0.110	69.405	1	<0.001	2.506	2.019–3.110
Ethnicity			4.778	2	0.092		
Han						1	
Uygur	-0.031	0.056	0.314	1	0.575	0.969	0.869–1.081
Kazakh	0.094	0.057	2.713	1	0.100	1.099	0.982–1.230
Smoking	0.122	0.057	4.600	1	0.032	1.130	1.011–1.264
Drinking	0.164	0.066	6.187	1	0.013	1.178	1.035–1.340
BMI			8.222	2	0.016		
Normal						1	
Overweight	0.114	0.053	4.701	1	0.030	1.121	1.011–1.242
Obese	0.141	0.054	6.734	1	0.009	1.152	1.035–1.282
Diabetes	0.104	0.084	1.520	1	0.218	1.109	0.941–1.309
Hypertension			32.435	3	<0.001		
IDH	0.338	0.078	18.973	1	<0.001	1.402	1.204–1.632
SDH	0.262	0.058	20.634	1	<0.001	1.300	1.161–1.456
ISH	0.198	0.069	8.136	1	0.004	1.219	1.064–1.396
Anti-hypertensive therapy	0.131	0.082	2.531	1	0.112	1.140	0.970–1.340
TG	0.194	0.051	14.370	1	<0.001	1.214	1.098–1.342
TC	0.006	0.050	0.013	1	0.909	1.006	0.912–1.110
HDL-c	0.081	0.047	2.898	1	0.089	1.084	0.988–1.189
LDL-c	0.121	0.046	6.986	1	0.008	1.128	1.032–1.234
BUN	0.038	0.014	7.013	1	0.008	1.039	1.010–1.069
Cr	0.004	0.001	18.549	1	<0.001	1.004	1.002–1.006
UA	-0.001	0.000	2.751	1	0.097	0.999	0.999–1.000
Constant	-2.360	0.157	226.537	1	<0.001	0.094	

CIMT, carotid intima-media thickness; BMI, body mass index; IDH, isolated diastolic hypertension; SDH, systolic-diastolic hypertension; ISH, isolated systolic hypertension; TG, triglycerides; TC, total cholesterol; HDL-c, high-density lipoprotein cholesterol; LDL-c, low-density lipoprotein cholesterol; BUN, blood urea nitrogen; Cr, creatinine; UA, uric acid.

In addition to BUN, Cr and UA as measurement data, other variables were used as classification variables in the multivariate unconditional logistic regression analysis. Assignment expressions of these variables are presented in Table B in S1 File. Using men as a reference, we found that women had a lower risk of developing abnormal CIMT (OR: 0.704, 95% CI: 0.631–0.785). Using the 35- to 44-year-old group as a reference, we found that the risk of abnormal CIMT gradually increased with age up to the >75-year-old group (45- to 54-year-old group: OR: 1.481, 95% CI: 1.317–1.665; 55- to 64-year-old group: OR: 1.975, 95% CI: 1.746–2.234; 65- to 74-year-old group: OR: 2.727, 95% CI: 2.377–3.129). However, participants in the >75-year-old group (OR: 2.506, 95% CI: 2.019–3.110) had a lower risk of abnormal CIMT than those in the 65- to 74-year-old group. Moreover, the participants who smoked (OR: 1.130, 95% CI: 1.010–1.264) or drank alcoholic beverages (OR: 1.178, 95% CI: 1.035–1.340) had a higher risk of abnormal CIMT than non-smokers and non-drinkers (P<0.05). The risk of abnormal CIMT grew with increasing BMI; it was 1.12-fold higher in overweight participants (OR = 1.121, 95% CI: 1.011–1.242) and 1.15-fold higher in obese participants (OR = 1.152, 95% CI: 1.035–1.282). High TG dyslipidemia (OR: 1.214, 95% CI: 1.098–1.342), high LDL-c dyslipidemia (OR: 1.128, 95% CI: 1.032–1.234), high BUN (OR: 1.039, 95% CI: 1.010–1.069), and high Cr (OR: 1.004, 95% CI: 1.002–1.006) were also risk factors. Additionally, we found that the risk of abnormal CIMT was 1.40-fold higher in the IDH group (OR: 1.402, 95% CI: 1.204–1.632), 1.30-fold higher in the SDH group (OR: 1.300, 95% CI: 1.161–1.456), and 1.22-fold higher in the ISH group (OR: 1.219, 95% CI: 1.064–1.396). However, ethnicity, diabetes, anti-hypertensive therapy, high TC dyslipidemia, low HDL-c dyslipidemia, and high UA were not risk factors for abnormal CIMT (P>0.05).

### Multivariate unconditional logistic regression analysis for risk factors for abnormal CIMT within the three ethnic groups

After finding differences in the prevalence of abnormal CIMT among the different ethnic groups, we applied multivariate unconditional logistic regression analysis on all identified risk factors and attempted to determine whether any differences in these risk factors could explain the difference in abnormal-CIMT prevalence among these ethnicities. A comparison was made between normal CIMT participants and abnormal CIMT participants, including carotid intimal thickening and plaque, in the Han, Uygur and Kazakh ethnic groups. The results are shown in Table 4. In the Han participants, women had a lower risk of developing abnormal CIMT than men (OR: 0.539, 95% CI: 0.449–0.647). In addition, higher risks of abnormal CIMT were observed for participants ≥55 years old (the 55- to 64-year-old group: OR: 1.677, 95% CI: 1.377–2.043; the 65- to 74-year-old group: OR: 1.990, 95% CI: 1.616–2.450; and the >75-year-old group: OR: 1.934, 95% CI: 1.408–2.656), who drank alcohol (OR: 1.268, 95% CI: 1.055–1.525), who were obese (OR: 1.282, 95% CI: 1.078–1.524), with diabetes (OR: 1.304, 95% CI: 1.041–1.634), with SDH (OR: 1.323, 95% CI: 1.100–1.590), and with Cr (OR: 1.004, 95% CI: 1.002–1.007). In Uygur participants, women had a lower risk of developing abnormal CIMT than men (OR: 0.770, 95% CI: 0.631–0.941). In addition, higher risks of abnormal CIMT were observed for participants ≥45 years old (the 45- to 54-year-old group: OR: 2.988, 95% CI: 2.323–3.845; the 55- to 64-year-old group: OR: 4.372, 95% CI: 3.392–5.635; the 65- to 74-year-old group: OR: 7.731, 95% CI: 5.837–10.240; and the >75-year-old group: OR: 6.549, 95% CI: 4.402–9.745), with SDH (OR: 1.426, 95% CI: 1.160–1.753), with ISH (OR: 1.844, 95% CI: 1.470–2.313), and with high TG levels (OR: 1.247, 95% CI: 1.044–1.489), high BUN (OR: 1.055, 95% CI: 1.006–1.107), and high Cr (OR: 1.004, 95% CI: 1.002–1.007). In the Kazakh participants, women had a lower risk of developing abnormal CIMT than men (OR: 0.813, 95% CI: 0.666–0.993). In addition, a higher risk of abnormal CIMT was observed in participants between 45–74 years old (the 45- to 54-year-old group: OR: 1.230, 95% CI: 1.003–1.508; the 55- to 64-year-old group: OR 1.336, 95% CI: 1.060–1.684; and the 65- to 74-year-old group: OR 2.004, 95% CI: 1.517–2.647), with elevated BMI (overweight: OR: 1.414, 95% CI: 1.154–1.733; and obese: OR: 1.340, 95% CI: 1.098–1.636), with IDH (OR: 1.536, 95% CI: 1.170–2.016), with high TC levels (OR: 1.217, 95% CI: 1.017–1.456), with low HDL-c levels (OR: 1.231, 95% CI: 1.027–1.476), and with high LDL-c levels (OR: 1.224, 95% CI: 1.035–1.446).

**Table 4:** Multivariate unconditional logistic regression analysis for risk factors for abnormal CIMT in the different ethnic groups.

	Han			Uygur			Kazakh		
	P	OR	95% CI	P	OR	95% CI	P	OR	95% CI
Gender	<0.001	0.539	0.449–0.647	0.011	0.770	0.631–0.941	0.043	0.813	0.666–0.993
Age	<0.001			<0.001			<0.001		
35–44		1			1			1	
45–54	0.077	1.184	0.982–1.429	<0.001	2.988	2.323–3.845	0.047	1.230	1.003–1.508
55–64	<0.001	1.677	1.377–2.043	<0.001	4.372	3.392–5.635	0.014	1.336	1.060–1.684
65–74	<0.001	1.990	1.616–2.450	<0.001	7.731	5.837–10.240	<0.001	2.004	1.517–2.647
>75	<0.001	1.934	1.408–2.656	<0.001	6.549	4.402–9.745	0.146	1.488	0.870–2.545
Smoking	0.949	0.994	0.831–1.190	0.182	1.189	0.922–1.533	0.509	1.064	0.886–1.277
Drinking	0.011	1.268	1.055–1.525	0.235	1.197	0.889–1.612	0.183	0.844	0.658–1.083
BMI	0.017			0.850			0.001		
Normal		1			1			1	
Overweight	0.647	1.037	0.886–1.215	0.607	0.950	0.783–1.154	0.001	1.414	1.154–1.733
Obese	0.005	1.282	1.078–1.524	0.664	0.957	0.785–1.166	0.004	1.340	1.098–1.636
Diabetes	0.021	1.304	1.041–1.634	0.526	0.904	0.660–1.236	0.778	0.941	0.618–1.433
Hypertension	0.010			<0.001			0.012		
IDH	0.053	1.238	0.997–1.536	0.065	1.417	0.979–2.051	0.002	1.536	1.170–2.016
SDH	0.003	1.323	1.100–1.590	0.001	1.426	1.160–1.753	0.157	1.162	0.944–1.431
ISH	0.722	1.042	0.832–1.305	<0.001	1.844	1.470–2.313	0.866	0.977	0.743–1.284
Anti-hypertensive therapy	0.782	0.968	0.771–1.216	0.633	1.103	0.737–1.652	0.071	0.911	0.822–1.008
TG	0.051	1.162	0.999–1.351	0.015	1.247	1.044–1.489	0.314	1.124	0.895–1.410
TC	0.128	0.891	0.769–1.034	0.219	0.882	0.723–1.077	0.032	1.217	1.017–1.456
HDL-c	0.616	0.964	0.836–1.112	0.122	1.145	0.964–1.359	0.024	1.231	1.027–1.476
LDL-c	0.453	1.055	0.917–1.215	0.076	1.163	0.984–1.374	0.018	1.224	1.035–1.446
BUN	0.506	1.016	0.969–1.066	0.029	1.055	1.006–1.107	0.098	1.050	0.991–1.112
Cr	0.001	1.004	1.002–1.007	0.002	1.004	1.002–1.007	0.629	0.999	0.994–1.004
UA	0.139	0.999	0.998–1.000	0.588	1.000	0.999–1.002	0.742	1.000	0.999–1.001

BMI, body mass index; IDH, isolated diastolic hypertension; SDH, systolic-diastolic hypertension; ISH, isolated systolic hypertension; TG, triglycerides; TC, total cholesterol; HDL-c, high-density lipoprotein cholesterol; LDL-c, low-density lipoprotein cholesterol; BUN, blood urea nitrogen; Cr, creatinine; UA, uric acid.

## Discussion

Atherosclerosis is the pathological basis of coronary heart disease, hypertension, and stroke. Atherosclerosis can develop in large and small arteries that supply a variety of end organs, including the heart, brain, kidneys, and extremities [2]. Carotid intimal thickening and carotid plaque are two stages of carotid atherosclerosis. The normal arterial wall is composed of an intima membrane layer, a media layer and an outer layer. In the process of atherosclerosis formation, the arterial intima is the first to develop a lesion from the fatty streak, consisting of lipid-laden foam cells, which are macrophages that have migrated as monocytes from the circulation into the subendothelial layer of the intima. Therefore, carotid intima thickening is an early sign of carotid atherosclerosis [17]. The CIMT is based on the thickness of 2 layers (the intima and media) of the wall of the carotid arteries. The fatty streak evolves into a fibrous plaque, consisting of intima smooth-muscle cells surrounded by connective tissue and intracellular and extracellular lipids. People who develop atherosclerosis have an abnormally elevated CIMT. Ultrasound has a great advantage in the detection of carotid atherosclerosis; it can detect plaques and determine the degree of narrowing in the arteries that may be caused by atherosclerosis [18]. In the Multi-Ethnic Study of Atherosclerosis (MESA), the detection of carotid plaque burden showed promise in improving CVD risk prediction. In addition, the association of coronary calcium prevalence and CIMT was largely consistent among different ethnicities. Black patients had the highest common CIMT, and Chinese patients had the lowest CIMT [19]. In our investigation, the mean common CIMT was 0.86±0.003 mm in the total group, and the CIMT was 0.92±0.005 mm in the male participants, which is higher than the CIMT of Chinese participants in the MESA study. In addition, Uygur participants had the lowest CIMT (0.82±0.006 mm) among the three ethnic groups. The CIMT was similar in the Han (0.88±0.005 mm) and Kazakh (0.88±0.005 mm) participants (P>0.05). Additionally, we investigated the prevalence of carotid atherosclerosis in Xinjiang, a northwestern region of China. The overall prevalence of carotid intimal thickening was 12.4%, and the incidence of carotid plaques was 9.7%. These results indicate the presence of ethnic differences in the prevalence of abnormal CIMT.

Arterial remodeling processes in hypertension and carotid atherosclerosis involve similar pathological changes, including the inflammatory response and damage caused to endothelial cells in the arterial vascular wall. Hypertension and atherosclerosis have a mutually reinforcing relationship [20]. The prominent pathological changes in hypertension are vascular remodeling and hypertrophy. In the gradual development of hypertension, atherosclerosis is an important cause of vascular remodeling in small blood vessels, which further damages the target organs. Meanwhile, the increase in fluid shear stress also accelerates atherosclerosis in large vessels. The consequence of hypertension is systemic arterial atherosclerosis. Elevated blood pressure is one factor that promotes atherosclerosis. In the NHANES (National Health and Nutrition Examination Survey) conducted from 2009 to 2012, the prevalence of hypertension among US adults was estimated to be 32.6% [2]. In a recent study, the prevalence of hypertension was reported as 44.3% in China and 37% in the western region of China. It is noteworthy that the treatment rate and control rate of hypertension in the western part of China were 26.7% and 5.3%, respectively, which are lower than those of other regions in China [7]. Xinjiang, in the northwestern part of China, is a remote and multiethnic region, containing a total of 47 ethnicities, of which 13 are minorities. The Han Chinese account for 40% of the total population, whereas minorities, especially the Uygur and Kazakh Chinese, collectively account for another 40% of the total population. These ethnic groups have unique lifestyles and live in unique natural environments, thus differing from the population in other parts of China. In our study, we found that the prevalence of hypertension was 40.1% in the Xinjiang adult population, and the highest prevalence of hypertension was up to 50.3% in the Kazakh participants. Previous studies have shown that the control of risk factors can reduce the occurrence of cardiovascular disease by 80% [21]. Therefore, effective management of blood pressure level and other risk factors in patients with carotid intimal thickening and plaque will help reduce the incidence of cardiovascular disease.

There have been differing opinions on whether the measurement of mean common CIMT in individuals with elevated blood pressure would improve cardiovascular risk prediction. A meta-analysis based on prospective cohort studies showed that common CIMT measurements do not improve cardiovascular risk prediction in individuals with elevated blood pressure [22]. However, a recent study showed that a 5-year change in SBP is independently associated with the progression of carotid atherosclerosis in the Chinese population [23]. In our study, we also investigated the correlation between CIMT and blood pressure levels. We found a significant positive association of CIMT and blood pressure level in the different ethnicities. In addition, different subtypes of hypertension were strongly associated with CIMT, with significant ethnic differences. These results imply that the development of carotid atherosclerosis is closely related to the development of hypertension.

Atherosclerosis is considered to be caused by long-term effects of a combination of risk factors in the arterial wall [24]. Our data also illustrated that hypertension is associated with a higher risk of abnormal CIMT. In addition to elevated blood pressure, we investigated other factors associated with carotid atherosclerosis. In the total participant group, we found that the risk factors for abnormal CIMT were >45 years old, smoking, drinking, elevated BMI, hypertension, high TG and LDL-c dyslipidemia, BUN, and Cr. Compared with men, women have a lower risk of carotid atherosclerosis. In addition, we did not observe any association of some factors, such as diabetes, anti-hypertensive therapy, high TC, and low HDL-c and UA, with abnormal CIMT. It is noteworthy that ethnicity was also not an independent risk factor for carotid atherosclerosis within the total population. However, CIMT-related risk factors varied within the different ethnic groups in our study. The risk factors for abnormal CIMT in the Han group were ≥55 years old, drinking, obesity, diabetes, SDH, and Cr. In the Uygur group, risk factors for abnormal CIMT included ≥45 years old, SDH, ISH, high TG hyperlipidemia, BUN, and Cr. However, in the Kazakh group, the participants who were 45–74 years old and those with elevated BMI, IDH, high TC, low HDL-c, and high LDL-c dyslipidemia had a higher risk of carotid atherosclerosis.

In our study, we found that gender was a risk factor for abnormal CIMT in the three ethnic groups. Women had a lower risk of developing abnormal CIMT than men. In contrast, Huang et al. investigated the prevalence of carotid plaque in Chinese patients and found that gender was a predictive factor for carotid plaque progression in women with stroke and myocardial infarction [25]. These results imply that men are more likely to suffer from carotid atherosclerosis and that women with carotid plaque are more likely to suffer from stroke and myocardial infarction. Age was a common risk factor for abnormal CIMT in the different ethnic groups. However, we found that the age group at risk for abnormal CIMT was younger in the Uygur and Kazakh groups than in the Han group. This result implies that CIMT measurements should be of a greater importance in Uygur and Kazakh individuals ≥45 years old.

We observed an association of unhealthy lifestyles with abnormal CIMT in this study. We found that smoking was a risk factor for abnormal CIMT among the total participant group. However, when stratified by ethnicity, smoking was not found to be a risk factor for abnormal CIMT in this study. This result is interesting. In the Atherosclerosis Risk in Communities (ARIC) study, researchers assessed the direct effects of smoking on the development of atherosclerosis by measuring CIMT. In 10,914 patients, the progression of atherosclerosis in smokers was increased by 50% compared with that in non-smokers [26]. In our study, we focused more on the risk factors for abnormal CIMT, not only on carotid plaque in participants with no previous cardiovascular events or stroke. However, we expected smoking to contribute to abnormal CIMT through the accumulation of other risk factors. Multivariate analysis in our study showed that drinking and increased BMI were risk factors for abnormal CIMT, but there were significantly different results within the different ethnic groups. Drinking was a risk factor for abnormal CIMT only in the Han group. Obesity was a risk factor for abnormal CIMT in the Han group, and elevated BMI was a risk factor for abnormal CIMT in the Kazakh group. However, different types of dyslipidemia were associated with the risk of abnormal CIMT in the different ethnic groups. High TG was associated with a higher risk of abnormal CIMT in the Uygur group. In the Kazakh group, high TC, low HDL-c and high LDL-c were risk factors for abnormal CIMT. In the Xinjiang region, people are accustomed to high-salt, high-sugar, and high-fat diets; thus, hypertension, diabetes, and cardiovascular diseases are common in these populations. Currently, the awareness, treatment and control rate of hypertension, diabetes and dyslipidemia are lower in Xinjiang than in other parts of China. Thus, health education and effective drug therapies should be strengthened.

Interestingly, we found that ethnicity was not an independent risk factor for abnormal CIMT (P>0.05). However, the associations of abnormal CIMT and the different subtypes of hypertension varied within the different ethnic groups. The ethnicity and risk factor associations for abnormal CIMT are as follows: the Han group with SDH; the Uygur group with SDH and ISH; and the Kazakh group with IDH. Therefore, CIMT measurements should be a focus in patients with certain subtypes of hypertension within certain ethnic groups. Although the prevalence of carotid atherosclerosis was significantly different among the three ethnicities, there were some shared risk factors for carotid atherosclerosis. SDH is a common risk factor for carotid artery plaque in the Han and Uygur participants. Therefore, screening for carotid atherosclerosis is of importance in Han and Uygur patients with SDH. However, Kazakh participants with IDH were prone to developing carotid atherosclerosis. Therefore, periodic examination of CIMT by B-mode ultrasonography and increased anti-atherosclerosis treatment in various stages for different ethnic groups affected by different subtypes of hypertension will help to delay the development of carotid atherosclerosis. In addition, such treatment will help to reduce the incidence of cardiovascular diseases and stroke. Additionally, our results demonstrated an association between Cr and abnormal CIMT in the Han and Uygur groups. BUN was also associated with abnormal CIMT in the Uygur group.

## Conclusion

Although ethnicity was not an independent factor for abnormal CIMT in Xinjiang, a northwestern province in China, the associations of abnormal CIMT and different subtypes of hypertension varied for different ethnic groups. Han participants exhibited an association with SDH, Uygur participants exhibited an association with SDH and ISH, and Kazakh participants exhibited an association with IDH as risk factors for carotid atherosclerosis. Different ethnic backgrounds had different sets of risk factors for abnormal CIMT.

## Supporting information

S1 File Table A. General characteristics of participants. Table B. Assignment expressions of risk factors of abnormal CIMT.
(DOCX)
