# Supplementary material for: Association between carotid atherosclerosis and different subtypes of hypertension in adult populations: A multiethnic study in Xinjiang, China
Source: PLoS One. 2017 Feb 15;12(2):e0171791. doi: 10.1371/journal.pone.0171791 (PMC5310908; doi:10.1371/journal.pone.0171791)
Supplement: S1 File — Table A. General characteristics of participants. Table B. Assignment expressions of risk factors of abnormal CIMT. (DOCX) [file pone.0171791.s001.docx]

**Supporting information**

**Table A. General characteristics of participants.**

| **Group** | **Han (n=5,757)** | **Uygur (n=4,767)** | **Kazakh (n=4,094)** |
| --- | --- | --- | --- |
| Age (years) | 52.47±12.71 | 50.70±12.98^*^ | 48.63±11.69^*^† |
| BMI (kg/m^2^) | 25.13±3.50 | 25.84±4.42^*^ | 26.56±4.76^*^† |
| Smoking (n, %) | 1767 (30.7%) | 845 (17.7%)^*^ | 203 (5.0%)^*^† |
| Drinking (n, %) | 1098 (19.1%) | 466 (9.8%)^*^ | 587 (14.3%)^*^† |
| SBP (mmHg) | 132.74±19.96 | 131.48±21.23^*^ | 140.35±25.12^*^† |
| DBP (mmHg) | 84.97±15.58 | 80.10±14.88^*^ | 88.33±19.69^*^† |
| FBG (mmol/L) | 5.34±1.78 | 4.94±1.66^*^ | 5.13±1.51^*^† |
| TG (mmol/L) | 1.72±1.45 | 1.64±1.22^*^ | 1.21±0.93^*^† |
| TC (mmol/L) | 4.69±1.08 | 4.36±1.13^*^ | 4.78±1.16^*^† |
| HDL-c (mmol/L) | 1.26±0.45 | 1.26±0.47 | 1.29±0.43^*^† |
| LDL-c (mmol/L) | 2.87±0.91 | 2.8±0.92 | 2.90±0.93 |
| BUN (mmol/L) | 4.94±1.47 | 5.24±1.71^*^ | 4.66±1.53^*^† |
| Cr (umol/L) | 75.83±26.14 | 71.42±29.68^*^ | 70.48±19.80^*^ |
| UA (umol/L) | 306.24±86.85 | 249.44±76.11^*^ | 259.82±78.74^*^† |

BMI, body mass index; SBP, systolic blood pressure; DBP, diastolic blood pressure; FBG, fasting blood glucose; TG, triglycerides; TC, total cholesterol; HDL-c, high density lipoprotein-cholesterol; LDL-c, low density lipoprotein-cholesterol; BUN, blood urea nitrogen; Cr, creatinine; UA, uric acid. *P<0.05 vs. the Han participants; † P<0.05 vs. the Uygur participants.

**Table B. Assignment expressions of risk factors of abnormal CIMT.**

| Variables | Assignment expressions of variables | | | | |
| --- | --- | --- | --- | --- | --- |
| Gender | Male=1 | Female=2 |  |  |  |
| Age | 35~44 years=1 | 45~54 years=2 | 55~64 years=3 | 65~74 years=4 | more than 75 years=5 |
| Ethnicity | Han=1 | Uygur=2 | Kazahk=3 |  |  |
| Smoking | Smoking=1 | No smoking=2 |  |  |  |
| Drinking | Drinking=1 | No drinking=2 |  |  |  |
| BMI | Normal=1 | Overweight=2 | Obesity=3 |  |  |
| Diabetes | Diabetes=1 | No diabetes=2 |  |  |  |
| Blood pressure | Normal=1 | IDH=2 | SDH=3 | ISH=4 |  |
| Anti-hypertension drugs | No=1 | Yes=2 |  |  |  |
| TG | Normal=1 | High TG=2 |  |  |  |
| TC | Normal=1 | High TC=2 |  |  |  |
| HDL-c | Normal=1 | Low HDL-c=2 |  |  |  |
| LDL-c | Normal=1 | High LDL-c=2 |  |  |  |

BMI, body mass index; SBP, systolic blood pressure; DBP, diastolic blood pressure; TG, triglycerides; TC, total cholesterol; HDL-c, high density lipoprotein-cholesterol; LDL-c, low density lipoprotein-cholesterol.
